# TCP Transcription Factors Interact With NPR1 and Contribute Redundantly to Systemic Acquired Resistance

**DOI:** 10.3389/fpls.2018.01153

**Published:** 2018-08-14

**Authors:** Min Li, Huan Chen, Jian Chen, Ming Chang, Ian A. Palmer, Walter Gassmann, Fengquan Liu, Zheng Qing Fu

**Affiliations:** ^1^Department of Biological Sciences, University of South Carolina, Columbia, SC, United States; ^2^Institute of Plant Protection, Jiangsu Academy of Agricultural Sciences, Nanjing, China; ^3^Division of Plant Sciences, C.S. Bond Life Sciences Center and Interdisciplinary Plant Group, University of Missouri, Columbia, MO, United States; ^4^Jiangsu Key Laboratory for Food Quality and Safety-State Key Laboratory Cultivation Base of Ministry of Science and Technology, Nanjing, China

**Keywords:** plant immunity, systemic acquired resistance, transcriptional regulation, NON-EXPRESSER OF PR GENES 1, TCP transcription factors, *PATHOGENESIS-RELATED* genes

## Abstract

In *Arabidopsis*, TEOSINTE BRANCHED 1, CYCLOIDEA, PCF1 (TCP) transcription factors (TF) play critical functions in developmental processes. Recent studies suggest they also function in plant immunity, but whether they play an important role in systemic acquired resistance (SAR) is still unknown. NON-EXPRESSER OF PR GENES 1 (NPR1), as an essential transcriptional regulatory node in SAR, exerts its regulatory role in downstream genes expression through interaction with TFs. In this work, we provide biochemical and genetic evidence that TCP8, TCP14, and TCP15 are involved in the SAR signaling pathway. TCP8, TCP14, and TCP15 physically interacted with NPR1 in yeast two-hybrid assays, and these interactions were further confirmed *in vivo*. SAR against the infection of virulent strain *Pseudomonas syringae* pv. *maculicola* (*Psm*) ES4326 in the triple T-DNA insertion mutant *tcp8-1 tcp14-5 tcp15-3* was partially compromised compared with Columbia 0 (Col-0) wild type plants. The induction of SAR marker genes *PR1, PR2*, and *PR5* in local and systemic leaves was dramatically decreased in the *tcp8-1 tcp14-5 tcp15-3* mutant compared with that in Col-0 after local treatment with *Psm* ES4326 carrying *avrRpt2*. Results from yeast one-hybrid and chromatin immunoprecipitation (ChIP) assays demonstrated that TCP15 can bind to a conserved TCP binding motif, GCGGGAC, within the promoter of *PR5*, and this binding was enhanced by NPR1. Results from RT-qPCR assays showed that TCP15 promotes the expression of *PR5* in response to salicylic acid induction. Taken together, these data reveal that TCP8, TCP14, and TCP15 physically interact with NPR1 and function redundantly to establish SAR, that TCP15 promotes the expression of *PR5* through directly binding a TCP binding site within the promoter of *PR5*, and that this binding is enhanced by NPR1.

## Introduction

TCP proteins, as plant specific transcription factors (TFs), are named after the first characterized members, TEOSINTE BRANCHED1 (TB1) in maize (*Zea mays*), CYCLOIDEA (CYC) in snapdragon (*Antirrhinum majus*) and PCF in rice (*Oryza sativa*) (Nicolas and Cubas, 2016). Based on the sequence of the TCP specific helix-loop-helix DNA binding domain, 24 *Arabidopsis* TCP members are divided into class I and class II groups (Cubas et al., 1999; Martín-Trillo and Cubas, 2010). Class I proteins prefer to bind the consensus element KHGGGVC (Davière et al., 2014), whereas class II proteins bind the GTGGNCCC consensus DNA sequence (Aggarwal et al., 2010). TCP proteins govern essential functions in developmental processes, including endo-reduplication, seed germination, internode length, leaf shape, and flower development (Kieffer et al., 2011; Uberti-Manassero et al., 2012; Resentini et al., 2015; Lucero et al., 2017). In addition to regulating developmental processes, accumulating experimental evidence also implies that TCP TFs play key functions in plant immunity. Being convergently targeted by effectors from multiple pathogens suggested TCP TFs function essentially in plant immunity (Mukhtar et al., 2011; Weßling et al., 2014). TCP TFs were subsequently found to interact with SUPPRESSOR OF rps4-RLD1 (SRFR1), a negative immune regulator, and contributed redundantly to effector-triggered immunity (ETI) (Kim et al., 2014). TCP TFs can also mediate the activity of phytohormone. For instance, TCP8, TCP9, and other TCP proteins were verified to coordinately regulate the expression of *ISOCHORISMATE SYNTHASE 1* (*ICS1*) which is responsible for pathogen-induced salicylic acid (SA) biosynthesis (Wang et al., 2015). Furthermore, Type III effector HopBB1 promotes disease susceptibility via targeting and degrading TCP14, which functions as a negative regulator of the jasmonic acid signaling pathway (Yang et al., 2017). In addition to separate functions in plant immunity or developmental processes, TCP TFs can also serve as a bridge to connect both responses. A recent study provided evidence that TCP15 connects the plant immune response with cell cycle progression by interacting with MODIFIER OF snc1-1 (MOS1) (Zhang et al., 2018).

Systemic acquired resistance (SAR) is an induced plant immunity which can be activated by pathogen infection or SA application (Fu and Dong, 2013). Pathogen infection induces the accumulation of SA which functions as an endogenous immune signal (Fu et al., 2012). SA is required for SAR, because blocking SA accumulation suppresses SAR induction (Gaffney et al., 1993). In *Arabidopsis*, the expression of the *PATHOGENESIS-RELATED* (*PR*) genes *PR1, PR2* (encoding β-1,3-glucanase), and *PR5* (encoding a thaumatin-like protein) are used as hallmarks for SAR because they maintain high expression levels during SAR (Ward et al., 1991; Uknes et al., 1992). After synthesis on the rough endoplasmic reticulum, small *PR* proteins (5–75 kDa) are secreted and targeted to vacuoles, or to the apoplast where bacterial pathogens are found (Dong, 2004; Edreva, 2005). PR proteins are associated with disease resistance because they exhibit anti-microbial functions both *in vitro* and *in vivo* (Ryals et al., 1996; Edreva, 2005; Breen et al., 2017).

*Arabidopsis* NPR1 is required for SAR and SA induced expression of *PR1, PR2*, and *PR5* (Cao et al., 1994, 1997). As a critical transcriptional regulatory node in SAR, NPR1 regulates the expression of 2,248 out of 2,280 SA responsive genes (Wang et al., 2006). NPR1 lacks a DNA binding domain but contains two protein-protein interaction domains, suggesting that NPR1 functions as a cofactor by interacting with TFs to regulate downstream gene expression (Fan and Dong, 2002; Rochon et al., 2006; Boyle et al., 2009). Indeed, it was found that NPR1 interacts with the TGA subclass of basic Leu zipper (bZIP) family TFs to regulate the expression of *PR1* (Fan and Dong, 2002). Induced SA upon pathogen infection results in cellular redox potential change, which triggers the reduction of cytosolic oligomeric NPR1 into monomeric NPR1 (Mou et al., 2003). Monomeric NPR1 proteins then enter the nucleus and interact with TGAs to facilitate the expression of *PR1* (Fan and Dong, 2002; Rochon et al., 2006).

Since *PR1, PR2*, and *PR5* are co-induced by SAR inducers, TGA TFs are thought to co-regulate their expression. However, there are no TGA binding sites (TGACGt/g, ACGTCA) (Jakoby et al., 2002) within the promoter of *PR5*. In addition to chemical evidence, genetic evidence also suggests separate regulators exist that regulate the expression of *PR1, PR2*, and *PR5*. First, *PR2* and *PR5*, but not *PR1*, were found constitutively expressed in *WRKY70* overexpression transgenic plants (Li et al., 2004). Second, only *PR1* mRNA levels were found to be reduced in *enhanced disease susceptibility 5-1* mutants, while the mRNA levels of *PR2* and *PR5* showed no apparent change (Rogers and Ausubel, 1997). All these data suggest that additional NPR1-interacting TFs are required to explain how NPR1 regulates the expression of *PR5*, and prompted us to screen for new NPR1-interacting TFs. Here, we show that TCP8, TCP14, and TCP15 interact with NPR1, and that they contribute redundantly to SAR establishment. TCP15 is shown to bind to the TCP binding site within the promoter of *PR5* and promote its expression. In addition, the binding ability of TCP15 to the *PR5* promoter was enhanced by NPR1.

## Materials and methods

### Plant materials

All mutants and transgenic lines were derived from *Arabidopsis* [(*Arabidopsis thaliana* (L.) Heynh.)] ecotype Columbia-0 (Col-0). Single T-DNA insertion mutants *tcp8-1* (CS875709), *tcp14-5* (CS458588), *tcp15-3* (CS68533) (Kim et al., 2014), and *tcp15-1* (CS875923) (Kieffer et al., 2011) were purchased from Arabidopsis Biological Resource Center (ABRC). Double mutants *tcp8-1 tcp14-5, tcp8-1 tcp15-3, tcp14-5 tcp15-3* and triple mutant *tcp8-1 tcp14-5 tcp15-3* were described before (Kim et al., 2014). The mutant *npr1-2* was described before (Cao et al., 1997). *Agrobacterium tumefaciens* (strain GV3101) mediated transformation was used to construct transgenic plants through the floral dipping method. Transgenic lines *pTA:TCP15-EYFP* and *35S:GFP* (Wang et al., 2015) were provided by Dr. Ai-wu Dong. The *Dex:Flag-TCP15* constructs were transformed into the Col-0 and *npr1-2* background to generate *Dex:Flag-TCP15/*Col-0 *and Dex:Flag-TCP15/npr1-2* transgenic plants. T3 homozygous transgenic lines were screened on 1/2 Murashige, and Skoog (MS) medium with 10 μM hygromycin B. Inducible FLAG-TCP15 protein expression level was verified by immunoblot. The point mutation in the *npr1-2* mutant was confirmed by restriction enzyme digestion (Cao et al., 1997).

### Growth condition and chemical treatments

Seeds were vernalized at 4°C for 3 days before growth. Soil-grown plants were placed in a growth chamber at 22°C with 60% humidity under 12 h light. For *in vitro* growth, surface sterilized seeds were grown on MS plates at 22°C with 50% humidity under 16 h light. The bacterial strains of *Pseudomonas syringae* pv. *maculicola* (*Psm*) ES4326 and *Psm* ES4326 carrying *avrRpt2* were grown on King's B (KB) medium under streptomycin and both streptomycin and tetracycline selection, respectively at 28°C. SA solutions were diluted from a 100 mM sodium salicylate (Sigma Aldrich) stock solution. Dexamethasone (DEX) solutions were diluted from a 30 mM stock solution dissolved in ethanol.

### Plasmid construction

Primers used to amplify gene-specific sequences are listed in Supplementary Table 1. Fragments used in all constructs were validated by DNA sequencing. The *Arabidopsis* transcription factor library was purchased form ABRC (CD4-89). The entire coding regions of *NPR1, TCP8, TCP14*, and *TCP15* were amplified by PCR with Phusion® DNA Polymerase (NEB). PCR products were subsequently introduced into the Gateway® (GW) entry vector pDONR207 (Clontech) using GW BP Clonase II Enzyme Mix (Invitrogen). Resulting entry clones were cloned into GW destination vector pDEST22 or pDEST32 using LR Clonase II Enzyme Mix (Clontech) to generate pDEST22-*TCP8*, pDEST22-*TCP14*, pDEST22-*TCP15*, and pDEST32-*NPR1*. The promoter sequences of *PR1* (2,380 bp), *PR2* (1,513 bp), *PR5* (1,000 and 500 bp), and *NPR1* (1,000 bp) were amplified by PCR and also introduced into pDONR207 by BP reactions. Resulting entry clones were remobilized into GW destination vector pLacZi (Pruneda-Paz et al., 2014) using the LR reaction to generate *pPR1:lacZ, pPR2:lacZ, pPR5:lacZ*, and *pNPR1:lacZ* constructs. To introduce site-specific mutations within the promoter of *PR5*, the *pPR5: lacZ* plasmid DNA was amplified with a pair of complementary primers with GCGGGAC to ATAAACT mutations. After digestion with Dpnl enzyme (NEB), the resulting PCR products were transformed into *Escherichia coli* (*E. coli*) strain TOP10 by electroporation. *TCP15* from pDONR207-*TCP15* was introduced into the GW compatible vector pTA7002_*Flag*-GW (Chen et al., 2017) to generate *Dex:Flag-TCP15* constructs. *TCP14* and *TCP15* from pDONR207-*TCP14* and pDONR207-*TCP15*, respectively, were cloned into the pEarlyGate201 destination vector to make transient expression constructs *35S:HA-TCP14* and 3*5S:HA-TCP15* by LR reaction. *TCP8* from pDONR207-*TCP8* was cloned into the pEarlyGate202 destination vector to make a transient expression construct *35S:Flag-TCP8*. The *35S:NPR1-GFP* construct generated with pCB302 binary vector was described before (Mou et al., 2003).

### Yeast two-hybrid (Y2H) assays

Bait plasmid pDEST32-*NPR1* was transformed into the *Saccharomyces cerevisiae* strain AH109 (MATa). Prey plasmids pDEST22-*TFs* were transformed into the *S. cerevisiae* strain Y187 (MATα). Y2H library screening was described before Ou et al., 2011). The pDEST22 and pDEST32 empty vectors were served as negative controls. After mating, healthy diploid yeast cells growing on the double dropout medium without leucine and tryptophan (control plates) were selected. The triple dropout medium lacking leucine, tryptophan, and histidine with 1 mM 3-amino-1,2,4-triazole (3-AT) was used as selective plates. Aliquots of 10 μl diploid yeast cell suspension were spotted on control and selective plates at a concentration of OD_600_ = 1, 0.1, and 0.01. The yeast transformation, mating, plasmid isolation and interaction test processes described in the Yeast Protocols Handbook (Clontech) were followed.

### Yeast one-hybrid assays

The *promoter DNA:lacZ* constructs were first digested with restriction enzyme NcoI (NEB), and resulting linearized constructs were subsequently integrated into the chromosome of *S*. *cerevisiae* strain YM4271 (MATa). The constructs of pDEST22-*TCP8*, pDEST22-*TCP14*, and pDEST22-*TCP15* were transformed into the yeast strain YU (MATα) (Pruneda-Paz et al., 2014). The pDEST22 empty vector was also included to serve as a negative control. After mating, healthy diploid yeast cells growing on the double dropout medium lacking tryptophan and uracil were selected. The binding ability of the prey transcription factors to the bait promoter was calculated with the β-galactosidase activity assay described previously (Zheng et al., 2015).

### *Agrobacterium*-mediated transient expression assay

The constructs *35S:HA-TCP14, 35S:HA-TCP15, 35S:HA-EV, 35S:Flag-TCP8, 35S:Flag-EV, 35S:NPR1-GFP, 35S:EV-GFP* and p19 were transformed into the *A. tumefaciens* strain GV3101. Resulting strains were grown in YEB culture with gentamicin, rifampicin, and kanamycin A at 28°C overnight. Bacterial cells were collected by centrifugation at 5,000 g for 5 min. Precipitated cells were washed twice and resuspended in induction buffer (10 mM MES, pH 5.7, 10 mM MgCl_2_, and 200 μM acetosyringone). A final concentration of resuspended cells at OD_600_ = 0.5 was used to infiltrate into young leaves of 4-week-old *Nicotiana benthamiana*. Infiltrated leaves were harvested at 48 h after infiltration and stored at −80°C for subsequent analysis.

### Plant protein extraction and immunoblotting

Aliquots of 0.15 g plant sample were homogenized with 150 μl protein extraction buffer [PEB, 50 mM Tris-HCl, pH 7.5, 150 mM NaCl, 5 mM EDTA, 0.1% Triton^TM^ X-100 (Sigma-Aldrich), 0.2% IGEPAL CA-630 (Sigma-Aldrich), 50 μM MG115 (Signa-Aldrich), 1 mM PMSF, 10 mM DTT, and 1 × protease inhibitor cocktail (Sigma-Aldrich)] using 2010 Geno/Grinder (SPEX). Total protein extracts were obtained by centrifuging homogenized culture at 15,000 g for 15 min twice at 4°C. Protein concentration was quantified with Bradford reagent (Bio-Rad) using a spectrophotometer (Eppendorf). Protein samples in 5 × sample buffer (250 mM Tris-HCl, pH 6.8, 500 mM DTT, 6% SDS, 0.08% bromophenol blue, and 30% glycerol) were denatured at 70°C for 15 min. After separation on a precast Express^TM^ PAGE gel (GeneScript) by electrophoresis, denatured protein samples were transferred onto a nitrocellulose membrane (GE Healthcare). Total protein was stained with Ponceau S solution (0.1% Ponceau S (Abcam) and 5% acetic acid) to verify equal protein loading. The membrane was first incubated with a primary antibody [anti-GFP (Clontech), anti-HA-peroxidase (3F10, Roche), or anti-FLAG M2 (Sigma-Aldrich)], then incubated with a secondary antibody [goat-anti-rabbit lgG-HRP (Agrisera) or goat-anti-mouse lgG-HRP (Santa Cruz Biotech)]. After incubation with chemiluminescent agent SuperSignal™ West Pico or Dura substrate (ThermoFisher), targeted proteins were visualized on photographic film using an SRX-101A Medical Film Processor (Konica).

### Co-immunoprecipitation (Co-IP)

NPR1-GFP with FLAG-TCP8, HA-TCP14, or HA-TCP15 were transiently co-expressed in *N. benthamiana*. After 48 h, 1.5 g *N. benthamiana* leaves were homogenized with PEB. Homogenized samples were centrifuged at 20,000 g for 30 min twice at 4°C. For the interaction of NPR1 with TCP14 or TCP15, protein extracts were incubated with 20 μl GFP-Trap®_ MA Beads (Chromotek) with gentle rotation at 4°C overnight. The conjugated beads were separated by a magnetic stand (Promega) and subsequently washed three times with cold wash buffer (10 mM Tris/HCl pH 7.5, 150 mM NaCl, and 0.5 mM EDTA). Beads were resuspended in 2 × Laemmli Sample Buffer (Bio-Rad). Immunoprecipitated proteins were eluted by boiling beads for 10 min. The bound HA-TCP14 and HA-TCP15 were detected by immunoblots with the anti-HA antibody. For the interaction between NPR1 and TCP8, protein extracts were incubated with 20 μl anti-FLAG M2 Magnetic Beads (Sigma-Aldrich) with gentle rotation at 4°C overnight. The bound NPR1-GFP proteins were detected by immunoblot with the anti-GFP antibody. No DTT was added to any buffer to prevent disrupting disulfide linkages in the anti-FLAG beads.

### Chromatin immunoprecipitation

Two grams of 12-day-old seedlings were used in each experiment. After treatment with 30 μM DEX for 24 h, samples were harvested at 24 h after treatment with 0.5 mM SA. The ChIP assays were performed according to a previously described protocol (Komar et al., 2016). For *pTA*:*TCP15-EYFP* and *35S:GFP* transgenic lines, chromatin was immunoprecipitated by Pierce^TM^ Protein A/G Magnetic Beads (ThermoFisher) bound with the anti-GFP antibody. For *Dex:Flag-TCP15/*Col-0 and *Dex:Flag-TCP15/npr1-2* transgenic lines, chromatin was immunoprecipitated by beads bound with the anti-FLAG M2 antibody. Primers used in the ChIP assays are listed in Supplementary Table 1.

### RNA extraction and quantitative PCR

RNA was extracted with TRIzol™ (Invitrogen) according to its protocol. cDNA was synthesized using reverse transcriptase (Quanta). Real-time quantitative polymerase chain reaction (RT-qPCR) was performed with SYBR® Green (Quanta). The expression level of *UBIQUITIN 5* (*UBQ5*) was used as an internal control. Three biological replicates were assayed. Primers for RT-qPCR are listed in Supplementary Table 1.

### SAR assay

Two lower leaves of 3-week-old plants were hand-infiltrated with 10 mM MgSO_4_ or avirulent pathogen *Psm* ES4326 carrying *avrRpt2* (OD_600_ = 0.02). Three days later, three upper leaves were hand-infiltrated with virulent pathogen *Psm* ES4326 (OD_600_ = 0.001). Leaf samples for bacterial growth were collected at 3 days after the secondary infection. Six plants were used in each treatment.

## Results

### TCP8, TCP14, and TCP15 interact with NPR1

**Figure 1:**
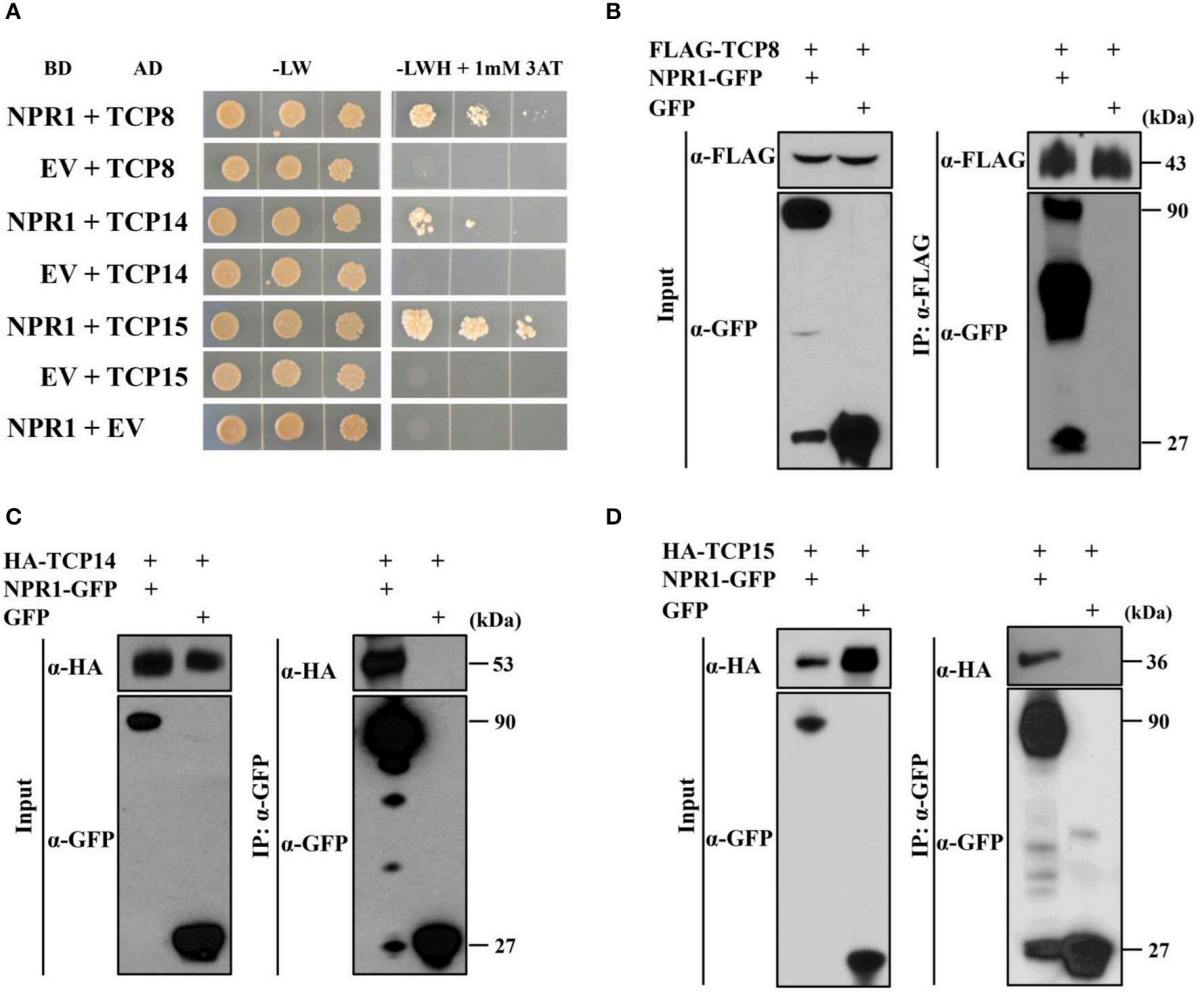
TCP8, TCP14, and TCP15 interact with NPR1. **(A)** Interactions between NPR1 with TCP8, TCP14, and TCP15 in yeast two-hybrid assays. Diploid yeast cells were serially diluted at OD_600_ = 1.0, 0.1, and 0.01. Aliquots of 10 μL of each dilution were plated on double synthetic dropout control plates without leucine and tryptophan (-LW) and selective triple synthetic dropout plates without leucine, tryptophan, and histidine with 1 mM 3-amino-1,2,4-triazole (–LWH + 1 mM 3AT). Photographs were taken 5 days after plating. EV, Empty vector; BD, GAL4 DNA-binding domain; AD, GAL4 activation domain. **(B)** Co-immunoprecipitation (Co-IP) of NPR1 with TCP8 in *Nicotiana benthamiana*. *NPR1-GFP* and *FLAG-TCP8* under control of the cauliflower mosaic virus (CMV) 35S promoter were transiently co-expressed in *N*. *benthamiana* by agroinfiltration. Total protein extracts were immunoprecipitated with anti-FLAG M2 magnetic beads. The input and immunoprecipitated protein were analyzed by immunoblot using the anti-FLAG and anti-GFP antibodies. kDa, kilodaltons. **(C,D)** Co-IP of TCP14 and TCP15 with NPR1 in *N*. *benthamiana*. Constitutively expressed *NPR1-GFP* was transiently co-expressed with *HA-TCP14* **(C)** and *HA-TCP15* **(D)** under control of the CMV 35S promoter in *N*. *benthamiana* by agroinfiltration. Total protein extracts were immunoprecipitated using anti-GFP trap beads. The input and immuno-precipitated proteins were analyzed by immunoblot using anti-HA and anti-GFP antibodies.

To test our hypothesis that NPR1 can interact with TFs other than TGAs, we performed Y2H screens (Ou et al., 2011) using *Arabidopsis* NPR1 as a bait and an *Arabidopsis* transcription factor library (Pruneda-Paz et al., 2014) as prey. Fifteen interactors were identified. To eliminate false positive interactions, we performed Y2H assays using individual candidate TFs as preys. Interactions were tested by the growth of diploid yeast cells on triple dropout medium lacking leucine, tryptophan, and histidine with 1 mM 3-AT. Supporting our initial assays, diploids containing NPR1 fused with a GAL4 DNA binding domain (BD-NPR1) and empty vector with a GAL4 activation domain (AD-EV) did not grow on selective plates (Figure 1A). Yeast diploids with BD-EV and AD-TFs were included as negative controls to exclude self-activation activity of TFs (Figure 1A). For this study, we focused on transcription factors in the TCP family. Yeast diploids containing BD-NPR1 and AD-TCP15 grew on selective plates (Figure 1A), indicating TCP15 was a true positive interactor of NPR1. We also found that yeast diploids containing BD-NPR1 with AD-TCP8 and AD-TCP14 grew on selective plates (Figure 1A), indicating NPR1 interacts with TCP8 and TCP14 in Y2H assays.

To test the association of NPR1 with TCP8, TCP14, and TCP15 *in planta*, we performed co-immunoprecipitation (Co-IP) assays in *N. benthamiana*. TCP8 fused with an N-terminal FLAG tag (FLAG-TCP8) and NPR1 fused with a C-terminal GFP tag (NPR1-GFP) were transiently co-expressed in *N. benthamiana* by agroinfiltration. NPR1-GFP proteins were co-immunoprecipitated with FLAG-TCP8 bound to the anti-FLAG magnetic beads (Figure 1B), indicating NPR1 associates with TCP8 *in planta*. NPR1-GFP and TCP14 fused with an N-terminal HA tag (HA-TCP14) (Figure 1C) or NPR1-GFP and HA-TCP15 (Figure 1D) were also transiently co-expressed in *N. benthamiana* by agroinfiltration. HA-TCP14 (Figure 1C) and HA-TCP15 (Figure 1D) proteins were co-immunoprecipitated with NPR1-GFP bound to the anti-GFP beads, indicating TCP14 and TCP15 are associated with NPR1 in *planta*. Together, results from Y2H and Co-IP assays indicate that TCP8, TCP14, and TCP15 physically interact with NPR1.

To investigate whether NPR1 affects the transcription of *TCP8, TCP14*, and *TCP15*, their mRNA levels in wild-type Col-0 and the *npr1-2* mutant after 24 h with or without SA application were examined by RT-qPCR. The SA-induced increase of *TCP8* mRNA levels was abolished in *npr1-2*, while the mRNA levels of *TCP14* and *TCP15* did not show a significant difference between *npr1-2* and Col-0 with or without SA for 24 h (Supplementary Figure 1). Our results indicate that NPR1 does not affect the transcription of *TCP14* and *TCP15* at this later time point, and an increase in SA-induced *TCP8* mRNA levels is dependent on NPR1.

### TCP8, TCP14, and TCP15 contribute redundantly to SAR establishment

**Figure 2:**
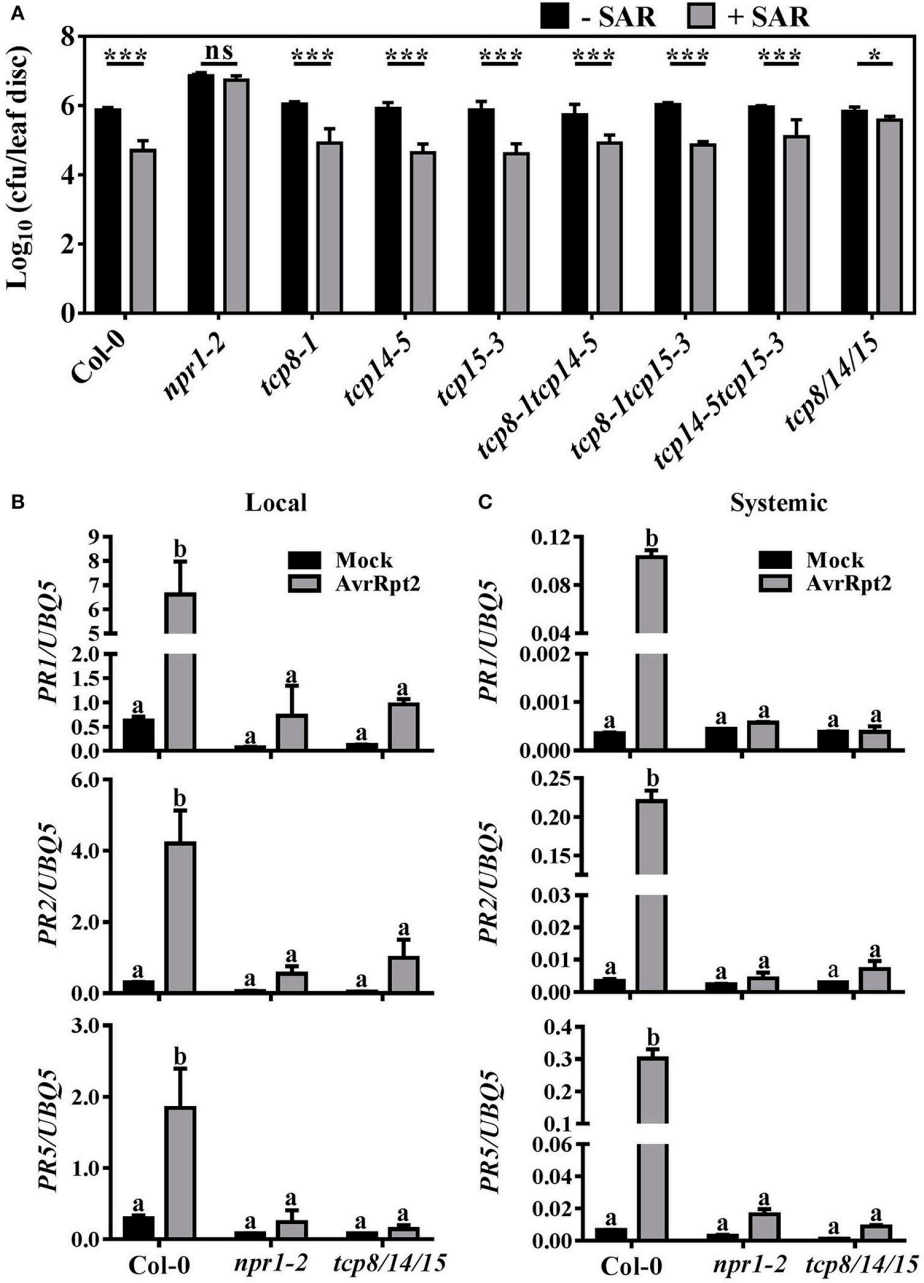
TCP8, TCP14, and TCP15 contribute redundantly to SAR establishment. **(A)** Bacterial population in Col-0, *npr1-2*, and the *tcp* mutants. Two lower leaves of 3-week-old plants were infiltrated with 10 mM MgCl_2_ (-SAR) or *Pseudomonas syringae* pv. *maculicola* (*Psm*) ES4326 carrying *avrRpt2* at OD_600_ = 0.02 (+SAR). Three days later, two upper leaves were infiltrated with *Psm* ES4326 at OD_600_ = 0.001. Bacterial growth in infected systemic leaves was calculated 3 days after the second infection. Error bars represent SD of six biological repeats. Statistical analysis was studied by *t*-test (*p < 0.05, ***p < 0.001) using Excel 2016. **(B,C)** The expression of *PR1, PR2*, and *PR5* in Col-0, *npr1-2*, and *tcp8/14/15*. Two local leaves were inoculated with 10 mM MgCl_2_ (Mock) or *Psm* ES4326 with *avrRpt2* at OD_600_ = 0.02 (AvrRpt2). The mRNA levels of *PR1, PR2*, and *PR5* in local leaves at 12 h post inoculation (hpi) **(B)** and in systemic leaves at 48 hpi **(C)** were analyzed by RT-qPCR. Values were normalized to the *UBQ5* mRNA levels. Error bars represent SD of three biological repeats. Statistical analysis was studied by two-way ANOVA following multiple comparisons with turkey test (95% confidence interval) using GraphPad Prism 7. Different small letters above the bars mean significant differences.

To investigate whether NPR1 interactors TCP8, TCP14, and TCP15 also function in SAR, we carried out SAR tests in the t*cp8-1, tcp14-5*, and *tcp15-3* single, corresponding double, and triple mutants (Kim et al., 2014). Two lower leaves were infected with *Psm* ES4326 carrying *avrRpt2* to induce SAR. Three days later, two upper leaves were infiltrated with the virulent pathogen *Psm* ES4326. The bacterial growth of *Psm* ES4326 in the *tcp8-1, tcp14-5*, and *tcp15-3* single and corresponding double mutants decreased 8.6- to 12.8-fold which was similar to that reduction (11.7-fold) in Col-0 after SAR induction (Figure 2A). These results demonstrated that the deletion of one or two of *TCP8, TCP14*, or *TCP15* genes is not enough to disrupt SAR. However, the *Psm* ES4326 population in *tcp8-1 tcp14-5 tcp15-3* (*tcp8/14/15*) only decreased 2.5-fold after SAR induction (Figure 2A), indicating that SAR induction was partially compromised in *tcp8/14/15*. Consistent with bacterial growth results, *Psm* ES4326 infected systemic leaves of the single and double mutants showed less chlorosis compared with that of *tcp8/14/15* after SAR induction (Supplementary Figure 2).

To examine whether the expression of SAR marker genes was affected, the mRNA levels of *PR1, PR2*, and *PR5* in local and systemic leaves of Col-0, *npr1-2*, and *tcp8/14/15* were examined by RT-qPCR assays. After locally treated with 10 mM MgCl_2_, the mRNA levels of *PR1, PR2*, and *PR5* in both local and systemic leaves of Col-0, *npr1-2*, and *tcp8/14/15* did not show a significant difference (Figure 2B), indicating TCP8, TCP14, and TCP15 did not affect the basal expression of *PR1, PR2*, and *PR5*. Whereas, after locally infected with *Psm* ES4326 with *avrRpt2*, the local and systemic induction of *PR1, PR2*, and *PR5* were all obviously decreased in *tcp8/14/15* compared with Col-0 (Figures 2B,C), consistent with the increased local susceptibility of *tcp8/14/15* to *avrRpt2*-expressing DC3000 (Kim et al., 2014). Together, these results reveal that TCP8, TCP14, and TCP15 contribute redundantly to establish SAR either directly or indirectly.

### TCP15 binds to the promoter of *PR5* at the TCP binding site

**Figure 3:**
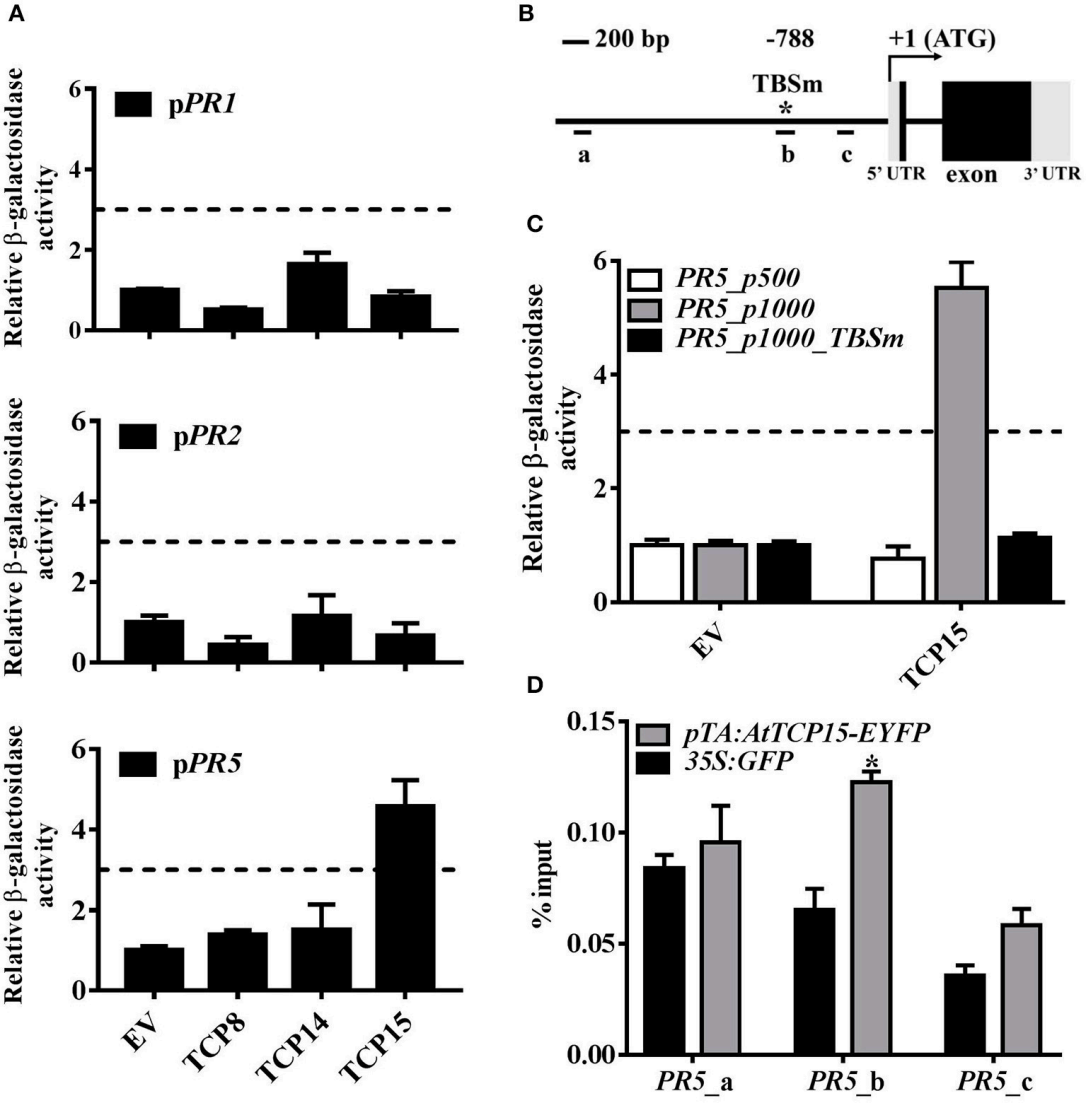
TCP15 binds to the *PR5* promoter at the TCP binding site. **(A)** Interactions between the TCP transcription factors and the *PR* promoters in yeast one-hybrid system (Y1H). Constructs expressing *pDEST22-TCPs* and empty *pDEST22* (EV) were transformed into the yeast strain YU. The *PR* promoters fused with *LacZ* reporter gene were integrated into the yeast strain YM4271. Healthy diploids grew on dropout medium without tryptophan and uracil were selected. Binding ability between TCPs and the *PR* promoters was quantified by β-galactosidase activity which was calculated in fold change over EV control. Three-fold induction was set as the cut-off. The binding ability of TCP8, TCP14, and TCP15 to the *PR1* promoter (upper panel), the *PR2* promoter (middle panel), and the *PR5* promoter (lower panel) is shown. Error bars represent SD of three repeats. **(B)** Diagram of the *PR5* gene structure. (+1) means transcription start site; long line indicates the promoter of *PR5*; short lines marked by a, b, and c represent the fragments amplified by RT-PCR in **(D)**; star shows TCP binding site located at −788 bp; TCPm represents TCP binding site with mutations. **(C)** Interactions between TCP15 with the *PR5* promoter containing a TCPm and the *PR5* promoter (500 bp) in Y1H assays. Error bars represent SD of three repeats. **(D)** Association between TCP15 and the *PR5* promoter was studied by chromatin immunoprecipitation (ChIP) assays combined with RT-PCR analysis. Twelve-day-old seedlings of *pTA:AtTCP15-EYFP* and *35S:GFP* transgenic plants were treated with 30 μM DEX for 24 h. Samples were collected at another 24 h after 0.5 mM SA induction. The enrichment of *PR5* promoter DNA in immunoprecipitated samples was shown as % input. Error bars represent SD of three technical repeats. Statistical analysis was studied by *t*-test (*p < 0.05) using Excel 2016. Another independent repeat showed similar results.

Since the reduction of mRNA levels of *PR1, PR2*, and *PR5* in *tcp8/14/15* may be a direct or indirect effect, we subsequently investigated whether TCP8, TCP14, and TCP15 can directly bind to the promoters of *PR1, PR2*, and *PR5* through yeast one-hybrid (Y1H) assays. Constructs of *pDEST22-TCPs* were transformed into the yeast strain YU, and *PR* promoters constructed in the pLacZi vector were integrated into the yeast strain YM4271 (Pruneda-Paz et al., 2014). Healthy yeast diploids growing on double dropout medium lacking tryptophan and uracil were selected. The binding affinity of TFs to promoters was quantified by β-galactosidase activity shown as fold change over empty vector control. In addition, 3-fold induction was set as the cut-off (Zheng et al., 2015). Expression of any TCP8, TCP14, or TCP15 with *PR1* and *PR2* promoters fused with the *LacZ* reporter gene did not activate more than a 3-fold change of β-galactosidase activity (Figure 3A), demonstrating that these TCP TFs did not bind to the promoter of *PR1* and *PR2*; however, expression of AD-TCP15 with the *LacZ* reporter gene fused *PR5* promoter resulted in a 4.13**-**fold change of β-galactosidase activity compared with vector control (Figure 3A), demonstrating that TCP15 physically bound to the *PR5* promoter and functioned as a transcriptional activator in yeast.

As a member of the class I TCP TFs, TCP15 was shown to bind to the consensus element KHGGGVC (Davière et al., 2014). Such a motif, GCGGGAC, was found in the promoter of *PR5* at −788 bp from the transcription start site (TSS) (Figure 3B). To investigate whether TCP15 targets the *PR5* promoter specifically at the TCP binding site (TBS), site mutations (GCGGGAC to ATAAACT) were introduced into the TBS (TBSm). The presence of TCP15 did not activate the expression of the *LacZ* reporter gene fused after the *PR5* promoter with TBSm (Figure 3C). To further confirm the binding specificity, a shorter *PR5* promoter (500 bp) without TBS was used. The presence of TCP15 did not activate reporter gene expression either (Figure 3C). These results indicate that the TBS within the promoter of *PR5* is required for the binding of TCP15 in yeast.

To further confirm this specific binding *in planta*, we performed chromatin immunoprecipitation (ChIP) assays using DEX inducible *TCP15* transgenic seedlings *pTA:AtCP15-EYFP* (Li et al., 2012). The relative enrichment of selected *PR5* promoter regions was tested by RT-PCR. Primers were designed to amplify three *PR5* promoter fragments a, b, and c, in which the b region contained the TBS (Figure 3B). Our data show that fragments a and c did not show obvious enrichment, while fragment b showed significant enrichment in the immunoprecipitated samples from *pTA:AtCP15-EYFP* compared with *35S:GFP* (Figure 3D), demonstrating that TCP15 binds *in vivo* to the TBS within the promoter of *PR5*. Our results from Y1H assays and ChIP assays indicate that TCP15 activates the transcription of *PR5* via binding to the TBS within its promoter.

### TCP15 regulates the expression of *PR5*

**Figure 4:**
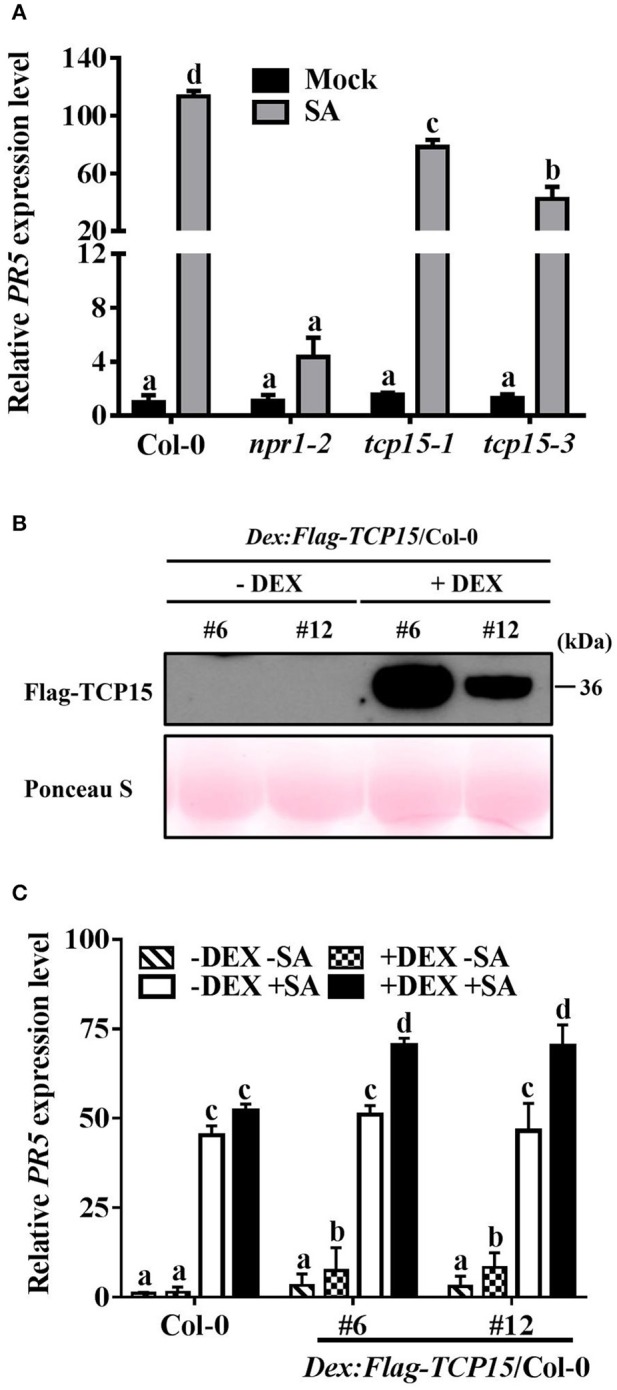
TCP15 regulates the expression of *PR5*. **(A)** The expression of *PR5* in Col-0, *npr1-2, tcp15-1*, and *tcp15-3*. Twelve-day-old seedlings were treated with water (Mock) or 0.5 mM SA (SA) for 24 h. The mRNA levels of *PR5* were analyzed by RT-qPCR. Values were normalized to the *UBQ5* mRNA levels, then to the value of Col-0 (mock), which was arbitrarily set at 1. Statistical analysis was studied by two-way ANOVA following multiple comparisons with turkey test (95% confidence interval) using GraphPad Prism 7. **(B)** FLAG-TCP15 protein levels in DEX inducible transgenic lines. Two T3 homozygous lines expressing *FLAG-TCP15* under control of a DEX-inducible promoter in the Col-0 background (*DEX:FLAG-TCP15*/Col-0) were used. Twelve-day-old seedlings were treated with either 0.01% ethanol (–DEX) or 30 μM DEX (+DEX) for 24 h. Protein levels were measured by immunoblot with the anti-FLAG antibody. Rubisco large subunits stained with Ponceau S were used to show equal total protein loading. **(C)** The expression of *PR5* in Col-0 and two *DEX:FLAG-TCP15*/Col-0 lines. Twelve-day-old seedlings described in **(B)** were treated with either 0.5 mM SA (+SA) or water (–SA) for 24 h after being treated with 0.01% alcohol (-DEX) or 30 μM DEX (+DEX) for 24 h. The *PR5* mRNA levels were examined by RT-qPCR. Values were normalized to the *UBQ5* mRNA levels, then to the value of Col-0 (–DEX –SA) which was arbitrarily set at 1. Error bars represent SD of three biological replicates. Statistical analysis was studied by two-way ANOVA following multiple comparisons with turkey test (95% confidence interval) using GraphPad Prism 7. Different small letters above the bars mean significant differences.

To investigate whether TCP15 affects the expression of *PR5*, the mRNA levels of *PR5* in Col-0, *npr1-2*, and the two *TCP15* T-DNA insertion lines *tcp15-1* (Kieffer et al., 2011) and *tcp15-3* were analyzed after SA treatment using RT-qPCR assays. The SA-induced *PR5* transcript level was significantly compromised in *tcp15-1* and *tcp15-3* compared with Col-0 (Figure 4A), indicating that TCP15 is required for SA-induced *PR5* expression.

To further confirm that TCP15 promotes the expression of *PR5*, the mRNA levels of *PR5* in Col-0, *npr1-2*, and two DEX inducible *TCP15* transgenic lines were analyzed after SA treatment using RT-qPCR assays. Since most constitutive overexpression *TCP15* transgenic lines showed growth arrest (Li et al., 2012), *Arabidopsis* transgenic lines expressing *FLAG-TCP15* under the control of a DEX-inducible promoter in the Col-0 background (*DEX:Flag-TCP15*/Col-0) were generated. Two T3 homozygous transgenic lines, #6 and #10, which exhibited different FLAG-TCP15 protein levels after DEX treatment, were used (Figure 4B). Before SA treatment, the presence of TCP15 in both transgenic lines significantly increased the mRNA levels of *PR5* (Figure 4C). With SA treatment, the presence of TCP15 increased the *PR5* mRNA levels to a dramatically higher level (Figure 4C), indicating that the up-regulation of *PR5* by TCP15 was promoted by SA. Taken together, these results indicate that TCP15 positively regulates the expression of *PR5*.

### NPR1 facilitates TCP15 binding to the *PR5* promoter

**Figure 5:**
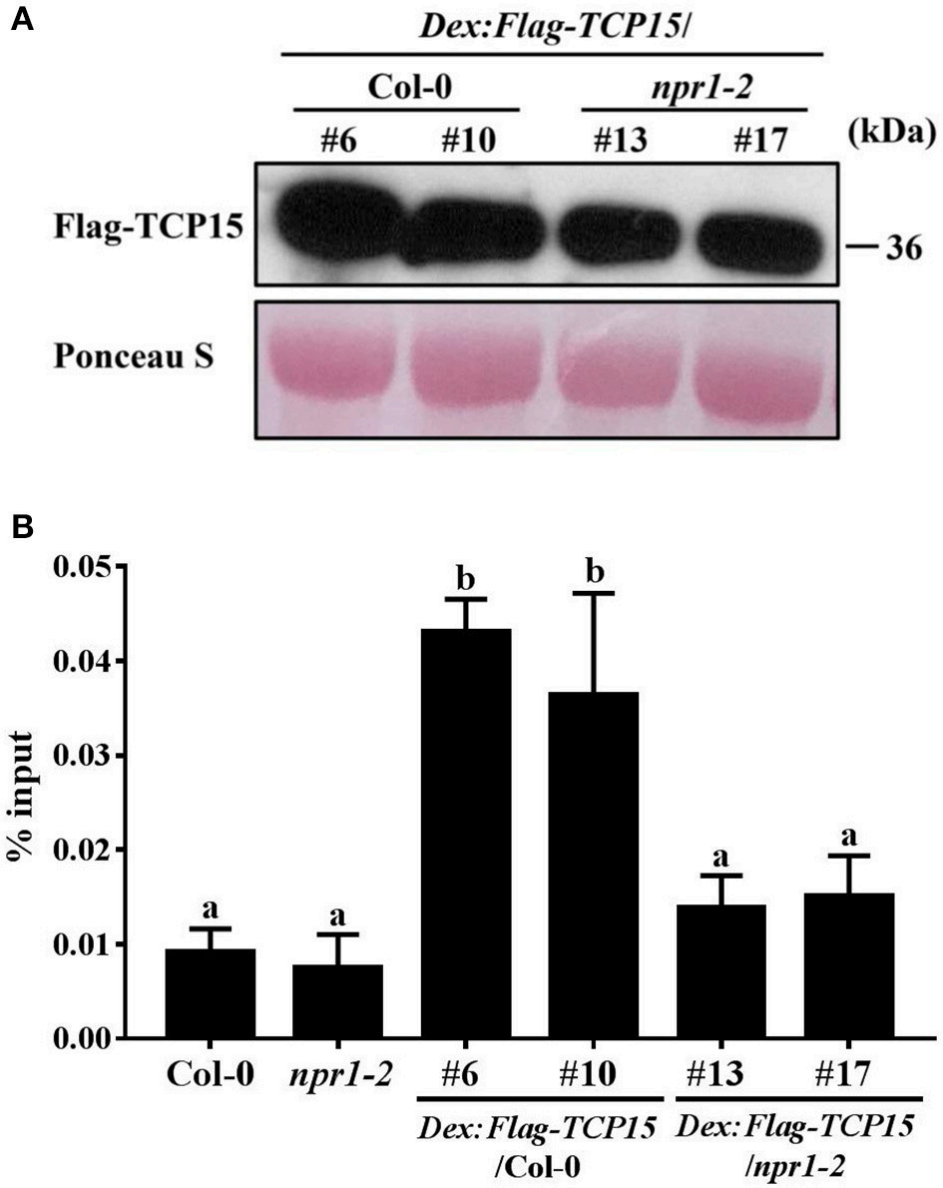
NPR1 enhances TCP15 binding to the *PR5* promoter. **(A)** FLAG-TCP15 protein levels in DEX inducible transgenic lines. Twelve-day-old seedlings of T3 homozygous lines expressing *FLAG-TCP15* under the control of a DEX-inducible promoter in the Col-0 and *npr1-2* background (*DEX:FLAG-TCP15*/*npr1-2*) were treated with 30 μM DEX for 24 h. Protein levels were measured by immunoblot with the anti-FLAG antibody. **(B)** The binding ability of TCP15 to the *PR5* promoter in transgenic plants described in **(A)** were examined with ChIP assays combined with RT-PCR analysis. Twelve-day-old seedlings were treated with 30 μM DEX, then treated with 0.5 mM SA after 24 h. Samples were collected 24 h after SA treatment. Primers amplifying fragment b described in Figure 3B were used in RT-PCR. The enrichment of *PR5* promoter DNA in immunoprecipitated samples was shown as % input. Error bars represent SD of three technical replicates. Statistical analysis was studied by two-way ANOVA following multiple comparisons with turkey test (95% confidence interval) using GraphPad Prism 7. Different small letters above the bars mean significant differences. Another independent replication demonstrated similar results.

Without a DNA binding domain, NPR1 functions as a transcriptional co-activator to facilitate TGA TFs binding to the promoter of *PR1* by interacting with TGA TFs (Zhang et al., 1999; Fan and Dong, 2002; Johnson et al., 2003). To test whether NPR1 can enhance TCP15 binding to the *PR5* promoter, we performed ChIP assays using *Arabidopsis* transgenic lines expressing *FLAG-TCP15* under the control of a DEX-inducible promoter in the Col-0 background and *npr1-2* background (*DEX:FLAG-TCP15*/*npr1-2*). The *DEX:FLAG-TCP15*/Col-0 transgenic lines #6 and #10 and *DEX:FLAG-TCP15*/*npr1-2* transgenic lines #13 and #17, which expressed similar Flag-TCP15 protein levels after DEX application (Figure 5A), were used. FLAG-TCP15 proteins and their cross-linked DNA were immuno-precipitated from chromatin extracts using beads bound with the anti-FLAG antibody. Immuno-precipitated DNA was analyzed by RT-PCR using primers amplifying fragment b described in Figure 3B. Fragment b showed significantly higher enrichment in *DEX:FLAG-TCP15*/Col-0 compared with *DEX:FLAG-TCP15*/*npr1-2* (Figure 5B), demonstrating that NPR1 facilitates TCP15 binding to the promoter of *PR5* in *Arabidopsis*.

## Discussion

In this work, we provide clear evidence that TCP8, TCP14, and TCP15 physically interact with NPR1 and coordinately contribute to SAR establishment, that TCP15 positively regulates the expression of *PR5* by directly binding to the TBS within the *PR5* promoter, and that NPR1 can enhance this binding. Our results suggest that in addition to acting as mediators of SA biosynthesis (Wang et al., 2015), TCP TFs are also essential players in the SA signaling pathway, and that TCP proteins not only contribute to ETI (Kim et al., 2014), but also function in SAR.

Taking advantage of the comprehensive *Arabidopsis* transcription factor library (Pruneda-Paz et al., 2014), TCP15 was identified as a novel NPR1 interactor through Y2H screens. Interactions between NPR1 and all the 24 *Arabidopsis* TCP proteins were also tested using Y2H assays, and more interactors were identified (unpublished data). As a critical transcriptional regulatory node, NPR1 directly regulates the expression of 2,248 SA-responsive genes (Wang et al., 2006). The absence of a DNA binding domain and the presence of two protein-protein interaction domains suggest NPR1 regulates the expression of downstream genes through interactions with TFs (Fu and Dong, 2013). Apparently, the known interactions between NPR1 and TGAs are not sufficient to explain the expression of all the genes regulated by NPR1. TCP proteins, as novel NPR1 interactors, will be excellent candidates to fill these missing links.

TCP proteins were reported to mediate SA biosynthesis by coordinately regulating the expression of *ICS1*, which encodes an enzyme responsible for pathogen-induced SA biosynthesis (Wang et al., 2015). This suggests that TCP proteins can affect the activity of NPR1. Although one conserved TCP binding motif was located at −169 bp from the TSS within the promoter of *NPR1*, no direct interaction was found between the *NPR1* promoter and TCP proteins in yeasts (Supplementary Figure 3), suggesting that TCP proteins could affect NPR1 activity indirectly. Because TCP8, TCP14, and TCP15 can interact with each other, and one characteristic of TCP TFs is their functional redundancy (Kim et al., 2014), it is not surprising that SAR could still be induced in the *tcp8-1, tcp14-5*, and t*cp15-3* single or corresponding double mutants (Figure 2A). SAR was abolished in the *npr1-2* mutants, while it was partially compromised in the *tcp8/14/15* triple mutants (Figure 2A), suggesting that other TFs are required in NPR1-mediated SAR signaling pathway. These TFs could be TGA proteins or other NPR1-interacting TCP proteins.

In addition, failed SAR induction could be caused by decreased initial immunity signal production and/or blocked mobile signal transduction. The expression of SAR marker genes (*PR1, PR2*, and *PR5*) in both local and systemic leaves were all significantly decreased after local infection with an avirulent pathogen (Figures 2B,C), consistent with a reduced initial immune signal production in *tcp8/14/15* plants (Kim et al., 2014). Our finding that TCP15 directly promotes the expression of *PR5* (Figures 4A,B) supports this explanation. As TCP8 and TCP14 did not bind the promoter of *PR5* in yeast, they may facilitate TCP15 binding to the promoter of *PR5* through a complex formation. Since TCP8, TCP14, and TCP15 did not show direct interactions with the promoter of *PR1* and *PR2* in Y1H assays (Figure 3A), the decrease of *PR1* and *PR2* may be indirectly regulated by these TCP TFs; however, whether TCP8, TCP14, and TCP15 are involved in SAR by mediating the activity of a mobile signal is unknown and will be an exciting topic to study in the future. Because *PR1* and *PR2* promoters both have TGA binding sites, it is possible that either TCP8, TCP14, and TCP15 interact with TGA transcription factors or the interactions between NPR1 and TCP8/14/15 promote the interactions between NPR1 and TGAs, to facilitate the expression of *PR1* and *PR2* to establish SAR.

How TCP15 responses to SA signaling and bind to the promoter of *PR5* is unclear. Based on the activity of TCP15 is dependent on redox modulation (Viola et al., 2013, 2016), the molecular mechanism of how SA induces the TCP15-dependent *PR5* expression is proposed. Under oxidizing conditions, the DNA binding activity of TCP15 is inhibited by dimers formed with disulfide bonds, while under reducing conditions induced by SA accumulation, the inhibition is reversed, resulting in extensively upregulated expression of *PR5*.

Although NPR1 could enhance TCP15 binding to the promoter of *PR5* (Figure 5B), the exact molecular mechanism through which NPR1 facilitates this association is unclear and will be an interesting topic to investigate. It is perhaps significant in this context that NPR1 activity is also regulated by SUMOylation-induced complex formation (Saleh et al., 2015), and TCP8, TCP14, and TCP15 were recently shown to associate with the nuclear SUMOylation machinery and to be SUMOylated as well (Mazur et al., 2017).

## Author contributions

ML, HC, JC, MC, WG, FL, and ZF contributed conception and design of the study. ML collected the data. ML, HC, WG, JC, and ZF organized the data. ML performed the statistical analyses. ML, IP, WG, and ZF wrote the manuscript. All authors contributed to manuscript revision, and have read and approved the submitted version.

### Conflict of interest statement

The authors declare that the research was conducted in the absence of any commercial or financial relationships that could be construed as a potential conflict of interest.
