# Supplementary material for: TCP Transcription Factors Interact With NPR1 and Contribute Redundantly to Systemic Acquired Resistance
Source: Front Plant Sci. 2018 Aug 14;9:1153. doi: 10.3389/fpls.2018.01153 (PMC6102491; doi:10.3389/fpls.2018.01153)
Supplement: Supplementary file 1 [file Data_Sheet_1.docx]

Supplementary Materials

TCP Transcription Factors Interact with NPR1 and Contribute Redundantly to Systemic Acquired Resistance

Min Li^1,2^, Huan Chen^1,2^, Jian Chen^1,2^, Ming Chang^1,2^, Ian Palmer^1^, Walter Gassmann^3^, Fengquan Liu^2,4^*, and Zheng Qing Fu^1^*

^1^Department of Biological Sciences, University of South Carolina, Columbia, SC, United States,

^2^Institute of Plant Protection, Jiangsu Academy of Agricultural Sciences, Nanjing, China,

^3^Division of Plant Sciences, C.S. Bond Life Sciences Center and Interdisciplinary Plant Group, University of Missouri, Columbia, MO, United States,

^4^Jiangsu Key Laboratory for Food Quality and Safety-State Key Laboratory Cultivation Base of Ministry of Science and Technology, Nanjing, China

*** Correspondence:**Fengquan Liu
[fqliu20011@sina.com](mailto:fqliu20011@sina.com)
Zheng Qing Fu
[zfu@mailbox.sc.edu](mailto:zfu@mailbox.sc.edu)

Keywords: plant immunity_1_, systemically acquired resistance_2_, transcriptional regulation_3_, NON-EXPRESSER OF PR GENES 1_4_, TCP transcription factors_5_, *PATHOGENESIS-RELATED* genes_6_.

# Supplementary Tables

**Supplemental Table 1. Primers used in this study**

| Primer Name | Primer Sequence (5'-3') |
| --- | --- |
| GW_F (attB1) | ggggACAAGTTTGTACAAAAAAGCAGGCTTC |
| GW_R (attB2) | gggcACCACTTTGTACAAGAAAGCTGGGTC |
| TCP8_GW_F | GW_F_ATGGATCTCTCCGACATCCG |
| TCP8_GW_R | GW_R_TCACTCAGAGCTATTTGAGTTCTCCT |
| TCP14_GW_F | GW_F_ATGCAAAAGCCAACATCAAGTATC |
| TCP14_GW_R | GW_R_CTAATCTTGCTGATCCTCCTCATCA |
| TCP15_GW_F | GW_F_ATGGATCCGGATCCGGATC |
| TCP15_GW_R | GW_R_CTAGGAATGATGACTGGTGCTTCC |
| PR1_p2380_GW_F | GW_F_ACAAAGAATATATATAACGATCATTG |
| PR1_p2380_GW_R | GW_R_AAAAATCGAGAATAGCCAGT |
| PR2_p1513_GW_F | GW_F_GCCGAACCAATATTCTATCAAG |
| PR2_p1513_GW_R | GW_R_TTTCTTGATTTTTCTATGATTCTGTG |
| PR5_p1000_GW_F | GW_F_GGTTCAATTTATGAACCAGTTTCTAC |
| PR5_p500_GW_F | GW_F_CAACAGTCAACAAACACATCTATCTG |
| PR5_p_GW_R | GW_R_ATTTTTTTATGGGTTTTTTGTGTG |
| NPR1_p1000_GW_F | GW_F_CCAAGAGGTGATTATGCAGA |
| NPR1_p1000_GW_R | GW_R_CAACAGGTTCCGATGAATTG |
| PR5_a_F | CTGTTGTTACACACACACACACCC |
| PR5_a_R | CCCCCTAATGAAGCATTCATCT |
| PR5_b_F | TCAACTATGTTCTAGACTTCTAGAAGCC |
| PR5_b_R | CTAAACAGGTATAAAATCTGGTTATAAACG |
| PR5_c_F | GGATCTCATAGTCGCGGATCA |
| PR5_c_R | CATGGCTGATGCACACTACACT |
| PR5_p1000_m_F | AACCCCCAGACAATAATATTTTATCGGTCA |
| PR5_p1000_m_R | TGACCGATAAAATATTATTGTCTGGGGGTT |

# Supplementary Figures

##
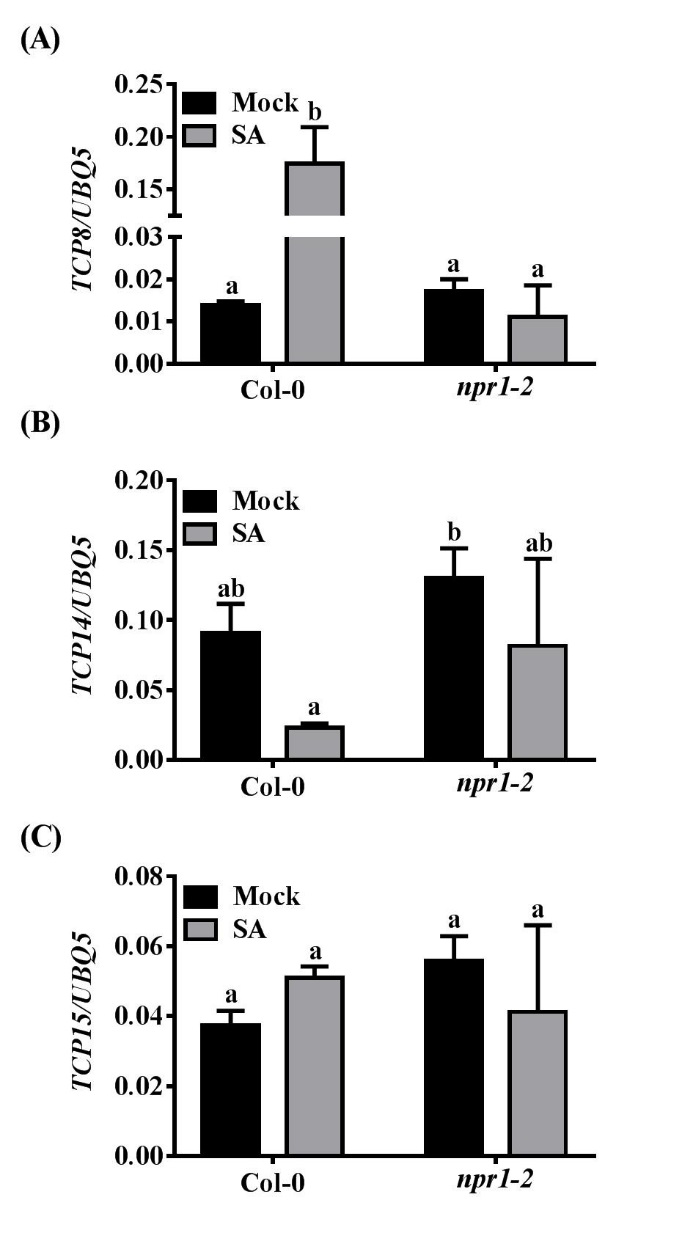


**Supplementary Figure 1.** SA-induced expression of *TCP8* requires *NPR1*.

Four-week-old plants were sprayed with water (Mock) or 0.5 mM SA (SA) for 24 hours. The mRNA levels of *TCP8* **(A)**, *TCP14* **(B),** and *TCP15* **(C)** in Col-0 and *npr1-2* were examined by RT-qPCR. Primers used in this study were described in (Wang et al., 2015). Relative mRNA levels were normalized to the *UBQ5* mRNA levels. Error bars represent SD of three biological repeats. Statistical analysis was studied by two-way ANOVA following multiple comparisons with turkey test (95% confidence interval) using GraphPad Prism 7. Different small letters above the bars means significant difference

**
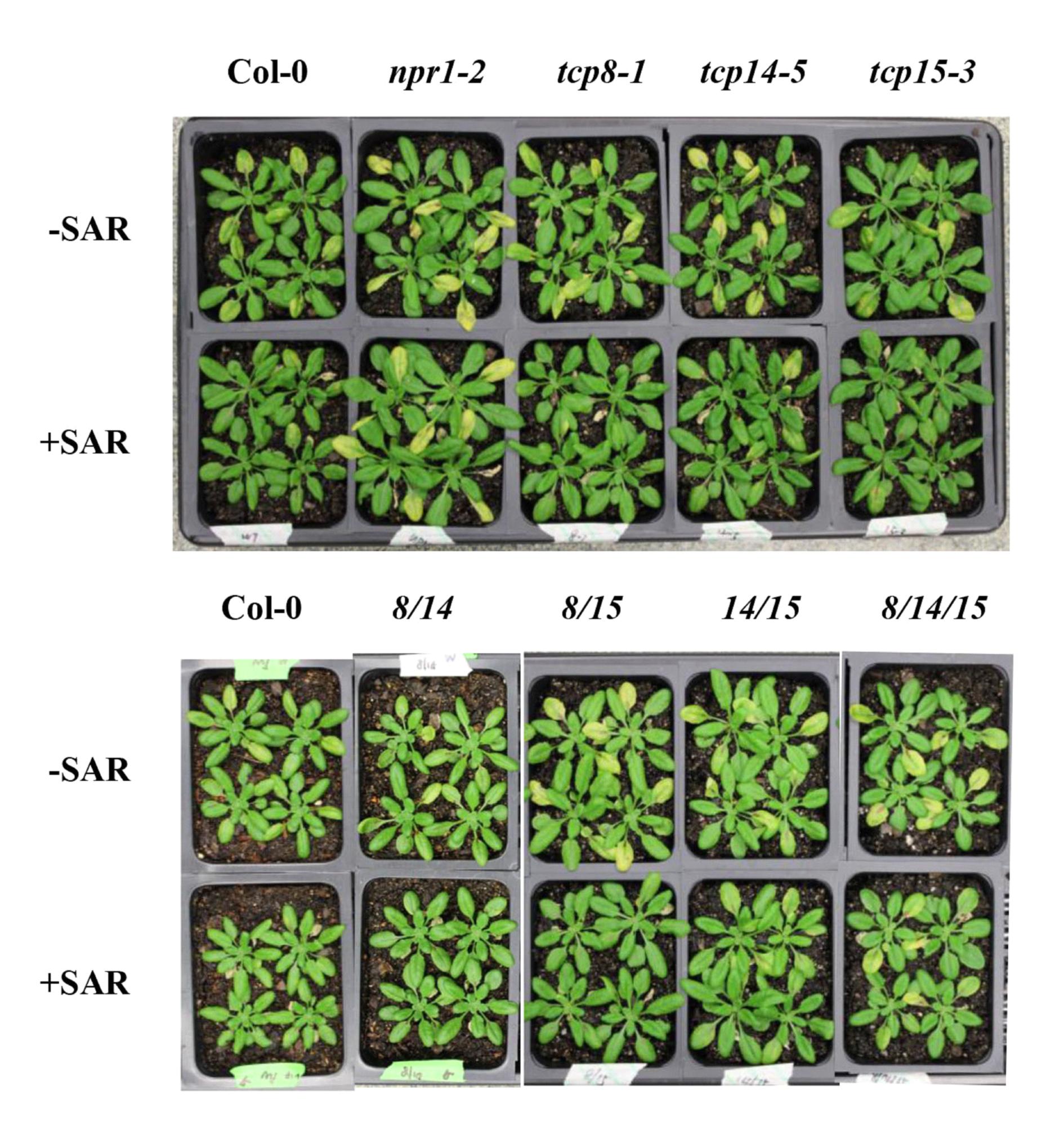
**

**Supplementary Figure 2.** The disease symptoms of plants subjected to SAR test.

Two lower leaves of three-week-old plants were infiltrated with 10 mM MgCl_2_ (-SAR) or *Psm* ES4326 with *avrRpt2* (OD_600_ = 0.02) (+SAR). Three days later, two upper leaves were infiltrated with *Psm* ES4326 (OD_600_ = 0.001). Photographs were taken three days post the second infection.

**
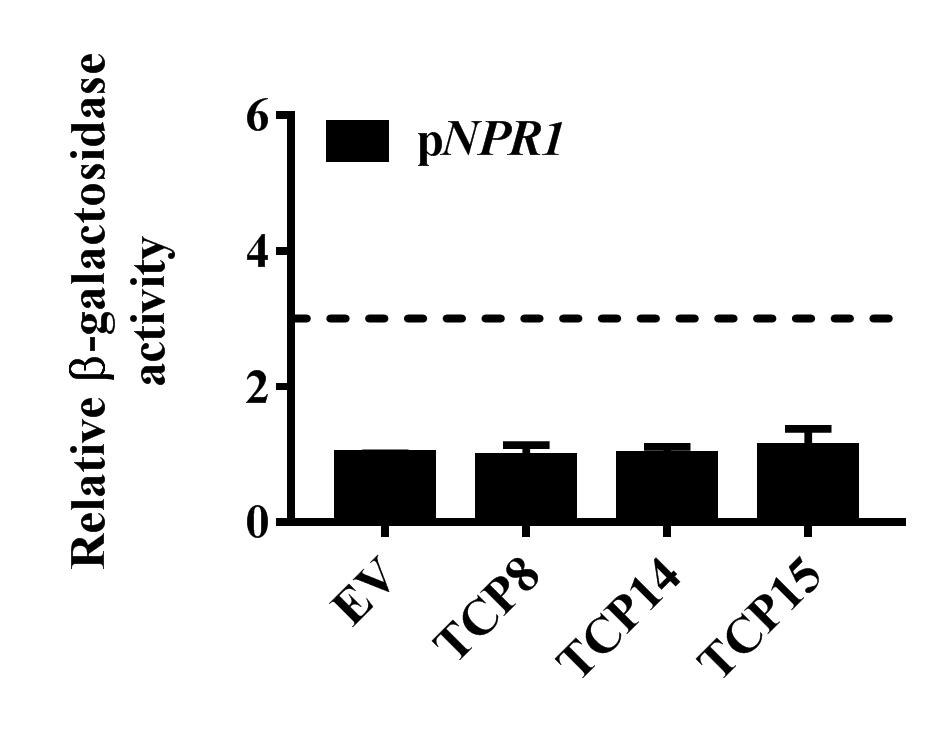
**

**Supplementary Figure 3.** TCP8, TCP14, and TCP15 did not bind to the *NPR1* promoter

The binding ability of TCP8, TCP14, and TCP15 to the *NPR1* promoter was studied by Y1H.
